# Systemic Delivery of Small Interfering RNA Targeting the Interleukin-2/15 Receptor β Chain Prevents Disease Progression in Experimental Arthritis

**DOI:** 10.1371/journal.pone.0078619

**Published:** 2013-11-05

**Authors:** Tiantian Zhang, Xuehua Bai, Xiaohua Mao

**Affiliations:** 1 Key Laboratory of Ministry of Education for Developmental Genes and Human Diseases, School of Life Sciences, Southeast University, Nanjing, China; 2 Department of Biochemistry, School of Medicine, Southeast University, Nanjing, China; INSERM-Université Paris-Sud, France

## Abstract

The role of interleukin (IL)-15 in the pathogenesis of rheumatoid arthritis (RA) is well established; however, systemic knockdown of IL-15 receptor (IL-15R) for reduction in inflammation at local sites has not been demonstrated. In this study, the therapeutic effect of intravenously administered siRNA targeting the β chain of IL-15R which is shared by the receptor for IL-2 was examined in rats with adjuvant-induced arthritis (AA). Polyethylenimine (PEI)-complexed siRNA nanoparticles could easily accumulate in arthritic paws of AA rats. In the paws, the nanoparticles were avidly taken up by macrophages and to a lesser extent by T cells. Weekly administered IL-2/15Rβ siRNA polyplexes were capable of decreasing disease progression in AA rats, with striking inhibition of clinical, radiologic, and histologic features of RA. The observed therapeutic effect was associated with reduced expression of proinflammatory mediators in the inflamed joints. Thus, this study provides evidence that IL-2/15Rβ could be targeted for the treatment of RA.

## Introduction

Rheumatoid arthritis (RA) is characterized by synovial hyperplasia and persistent inflammation that is now recognized to result from the interaction among macrophages, T cells, B cells, and nonhematopoietic cells such as fibroblasts. These interactions are facilitated by the actions of cytokines released from the activated cells that then, through both autocrine and paracrine mechanisms, induce the production of other proinflammatory cytokines, which together contribute to the pathogenesis of this disease and ultimately lead to joint damage [1], [2]. Accordingly, biologic agents that block the actions of specific cytokines or immunue regulators have emerged as major therapies in RA. However, no final victory has been achieved over this joint damaging and potentially life-threatening systemic autoimmune disease, as the effects of current biologics are only partial and nonresponses are common. A plausible explanation for variable response to the biologic agents is that distinct cytokines/immune regulators may mediate discrete effects at different disease stages, and thus, there may be optimal targets defined by the relative disease stage of intervention [1].

Based on the work pioneered by McInnes *et al*. [3], [4], the importance of IL-15 in the development and amplification of the inflammatory process in RA has been demonstrated. In RA, IL-15 is expressed primarily by macrophages as well as by fibroblast-like synoviocytes and endothelial cells [5]. It exhibits pleiotropic proinflammatory effects on numerous target cell types relevant to a variety of inflammatory conditions [6] and was thought to be at the apex of the cytokine cascade created in the inflamed joints [7]. This notion was strengthened by the fact that TNFα blockade does not affect synovial expression of IL-15 whereas neutralizing IL-15 significantly inhibits the production of proinflammatory cytokines including TNFα in RA synovial cell cultures [8], [9]. Most importantly, IL-15 plays a key role in sustaining the fundamentally important cognate interactions between T cells and macrophages which are involved in the production and release of most proinflammatory cytokines, chemokines and metalloproteinase enzymes [1], [6]. In clinical studies, the link between serum IL-15 levels and disease severity in patients with early arthritis has been demonstrated [10]; in addition, genetic variants in IL-15 were shown to associate with joint destruction in RA in a multicohort study, suggesting a direct role for this cytokine in articular bone erosion [11], [12]. As IL-15 is implicated in initiation and perpetuation of the inflammation and mediates osteoclastogenesis in RA, IL-15 signaling pathway could possibly be targeted therapeutically. In support of this view, several IL-15- or IL-15 receptor (IL-15R)-directed monoclonal antibodies and fusion proteins have been effective in ameliorating RA in animal models [13]–[15].

Due to their short plasma half lives, protein therapeutics for RA should be administered frequently over a long-term period, which may cause unwanted systemic side effects and safety complications. It is reported that siRNA-mediated gene silencing can last for several weeks in non-dividing cells such as tissue macrophages [16]. In this study, the therapeutic efficiency of a systemically delivered siRNA targeting the IL-15R β chain which is also shared by IL-2 receptor was assessed in experimental arthritis. We speculate that IL-2/15Rβ siRNA formulated with polyethylenimine (PEI) would be avidly taken up by inflamed macrophages, inhibit the production and release of most proinflammatory cytokines and thus prevent disease progression in rats with adjuvant-induced arthritis (AA).

## Results

### Design of siRNA against rat IL-2/15Rβ

**Figure 1:**
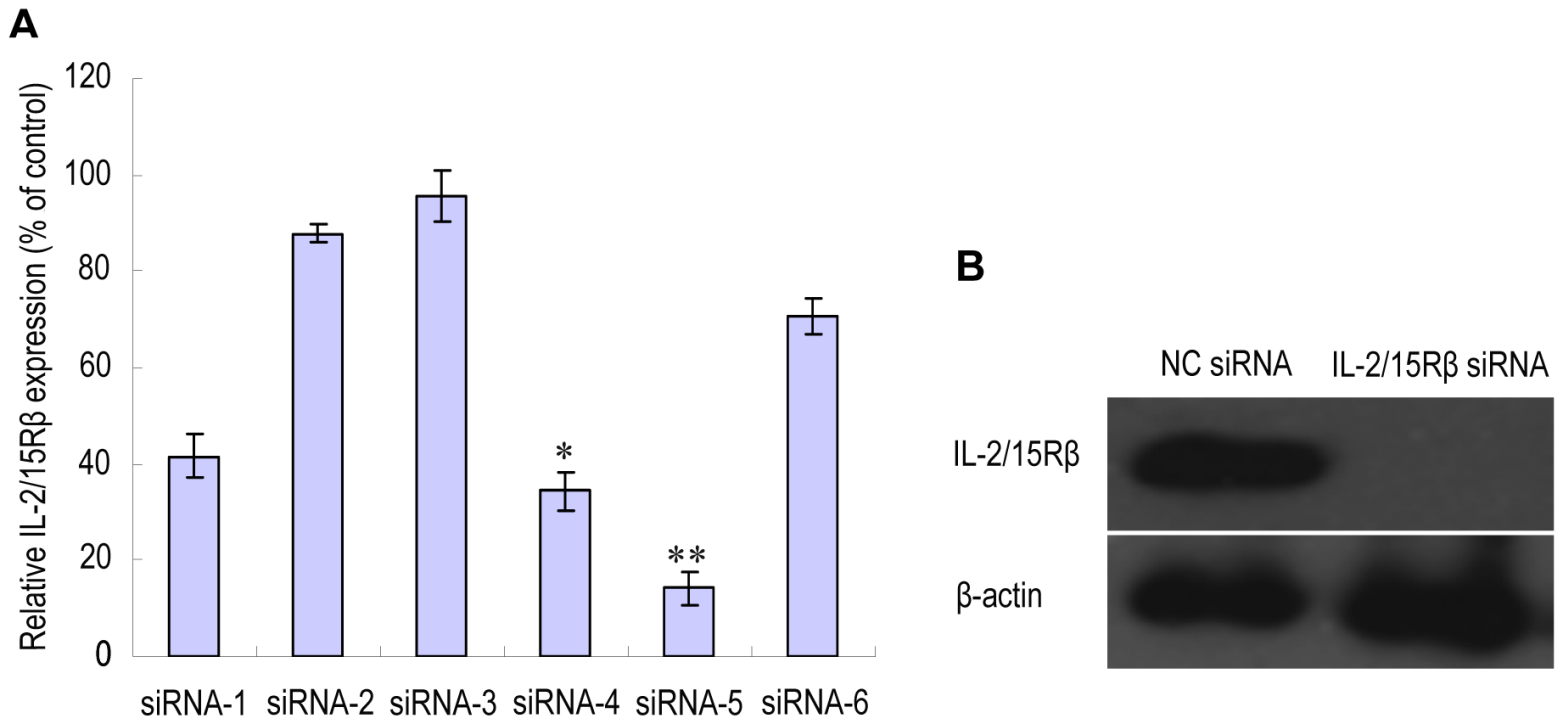
*In vitro* validation of siRNA sequences designed to target rat IL-2/15Rβ. Six different IL-2/15Rβ siRNA sequences were designed and rat peritoneal macrophages were transfected with each of the six siRNA duplexes or with a nontargeting negative control siRNA (NC siRNA) using X-tremeGENE siRNA transfection reagent (Roche). RNA and proteins were prepared at 24 and 48 h post-transfection, respectively. (A) IL-2/15Rβ mRNA levels measured by quantitative real time-PCR (qPCR). Results were normalized to GAPDH and are presented as the percentage of NC siRNA. *P<0.05; **P<0.01 versus NC siRNA. (B) Western blot analysis of IL-2/15Rβ protein levels in macrophages transfected with siRNA-5. The detection of actin expression was performed to monitor protein loading.

IL-15 is a cytokine that binds a heterotrimeric receptor containing a unique α chain (IL-15Rα), a β chain that is also found in the receptor for IL-2, and γ_c_ [17]. IL-15Rα binds IL-15 with high affinity, but transduces signals only in the presence of the IL-2/15Rβ and γ_c_. Since γ_c_ is shared by receptors for IL-4, IL-7, IL-9, IL-15, and IL-21 and a loss of γ_c_ function causes immunodeficiencies in both humans and mice [18], it is anticipated that knockdown of γ_c_ may lead to severe systemic side effects. In addition, it is unknown whether certain cytokines such as IL-4 and IL-9 play an inflammatory role in RA. In view of the well-established role of IL-15 and possible involvement of IL-2 in RA pathogenesis [19], [20], we attempted to test the feasibility of *in vivo* silencing of IL-2/15Rβ for the treatment of this disease. For this purpose, six siRNA sequences targeting rat IL-2/15Rβ were designed. Peritoneal macrophages were transfected with each of the six siRNA duplexes and the silencing of IL-2/15Rβ mRNA was measured by quantitative RT-PCR (qPCR). As shown in Figure 1A, the most potent effect was observed with siRNA-5, which successfully resulted in an 80% mRNA reduction relative to nonspecific negative control siRNA (NC siRNA). The silencing effect of siRNA-5 was further confirmed by Western blotting (Figure 1B). In the following experiments, siRNA-5 was used as IL-2/15Rβ-specific siRNA.

### Preparation and properties of PEI/siRNA complexes

**Figure 2:**
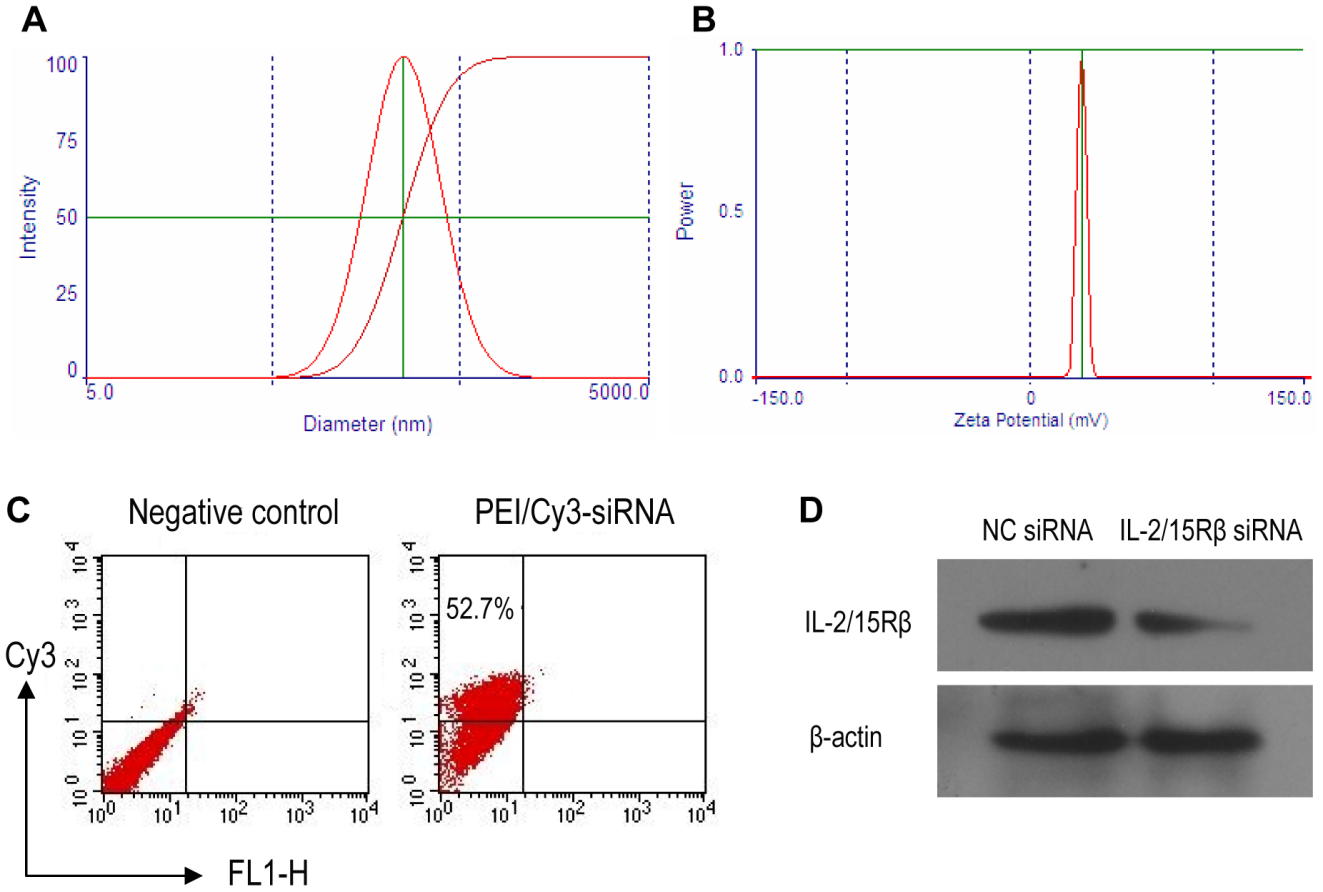
Physical and biological characterization of PEI/siRNA complexes. siRNA was complexed with *in vivo*-jetPEI at N∶P ratio of 6. (A) Hydrodynamic size distribution, and (B) zeta potential of PEI/NC siRNA complexes analyzed by DLS. (C) Flow cytometric analysis of the uptake of siRNA polyplexes in rat peritoneal macrophages. Cells were incubated with PEI-formulated Cy3-siRNA (40 nM) for 12 h. As negative control, cells were incubated with PEI/NC siRNA. (D) Western blot analysis of IL-2/15Rβ silencing in rat peritoneal macrophages. Macrophages were transfected with PEI/IL-2/15Rβ siRNA complexes for 48 h.

*In vivo*-jetPEI, a linear, endotoxin-free, cationic polymer currently used for the delivery of therapeutic nucleic acids in clinical trials was chosen as siRNA carrier. PEI/siRNA complexes were prepared by mixing *in vivo*-jetPEI with siRNA at N∶P ratio of 6 at which the PEI could tightly bind siRNA as analyzed by gel retardation assay (not shown). The resulting PEI/siRNA complexes displayed an average hydrodynamic diameter of about 246 nm and an average zeta potential of +28 mV as determined by DLS (Figures 2A, B). To examine the uptake of PEI/siRNA polyplexes by macrophages, PEI-formulated Cy3-siRNA (N∶P = 6) was incubated with rat peritoneal macrophages in the extracellular medium for 12 h. Next, intracellular uptake was analyzed by flow cytometry. As shown in Figure 2C, about 53% of the particles were internalized. Western blot analysis revealed that IL-2/15Rβ siRNA polyplexes were capable of knocking down IL-2/15Rβ expression in rat peritoneal macrophages by 60% compared to negative control siRNA (Figure 2D). The silencing capability of the PEI/IL-2/15Rβ siRNA complexes also indicated that IL-2/15Rβ siRNA could be released from the endocytosis vesicles.

### Tissue distribution of systemically administered PEI/siRNA complexes

**Figure 3:**
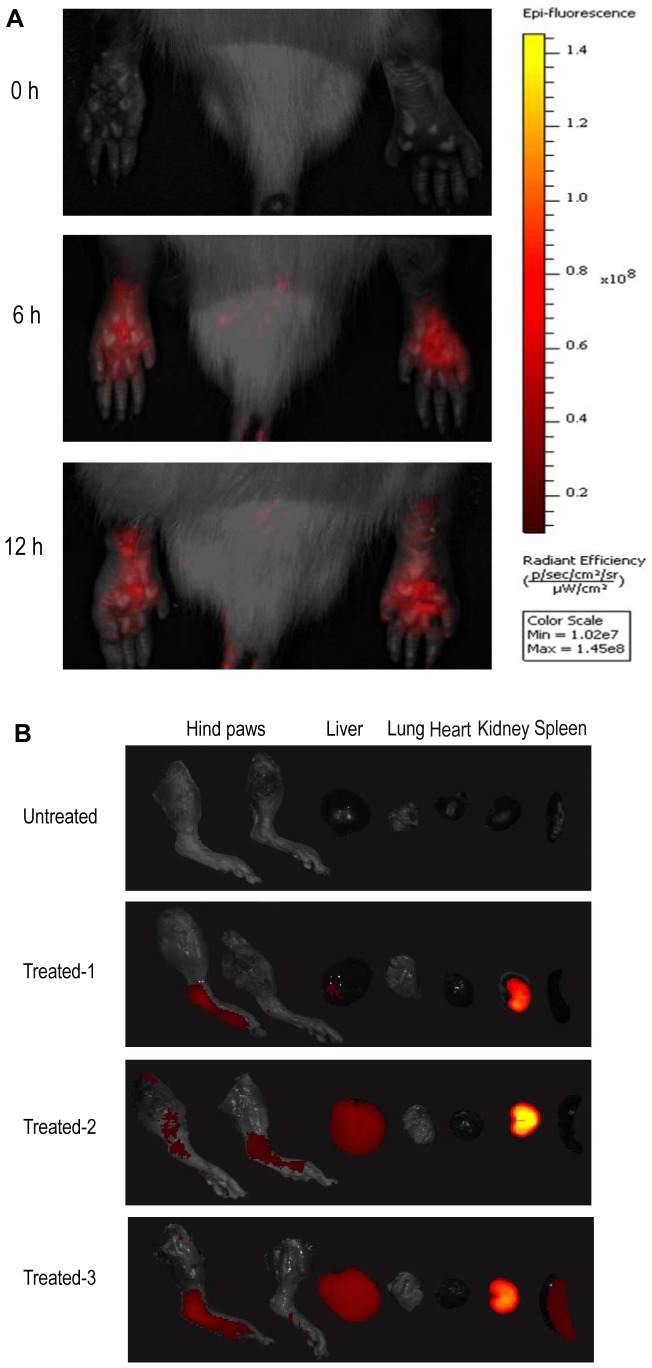
Tissue distribution of PEI/Cy5-siRNA polyplexes. The particles were administered as a single dose (0.6 mg siRNA/kg) to AA rats via tail vein. Images were taken with IVIS imaging system. 0 h represents the non-injected control. (A) *In vivo* fluorescence images of the arthritic paws at 6 and 12 h post-injection. Shown are representative of 3 rats per group. (B) Fluorescence images of major organs. Rats were killed 12 h after injection. Hind ankle joints and various organs (heart, liver, spleen, lung, and right kidney) from three rats were isolated and imaged.

Nanoparticles are usually taken up by liver, spleen and other parts of the reticuloendothelial system (RES). On the other hand, small particles can easily penetrate kidney's filtering systems. To determine tissue uptake and kinetics of PEI/siRNA complexes in the context of the inflammatory disorder, Cy5 was used to monitor the *in vivo* distribution of systemically applied PEI/siRNA nanoparticles. The long-wavelength emission spectrum of this fluorophore renders it distinguishable from background autofluorescence and thus very suitable for *in vivo* imaging [21]. AA rats were injected through the tail vein with a single dose of Cy5-siRNA formulated with the *in vivo*-jetPEI and tissue distribution was visualized at 6 and 12 h using Caliper IVIS imaging system. As shown in Figure 3A, Cy5-siRNA could accumulate in arthritic paws, and the accumulation pattern was similar at 6 h and 12 h after intravenous injection. To examine the distribution in detail, rats were sacrificed at 12 h after whole animal assay, and major organs including joints (without skin) were isolated. As shown in Figure 3B, the kidneys were found to possess the strongest signals among all tissues examined, whereas the liver, spleen, and joint displayed background to moderate signals. Note that the signal intensity in the excised joints was weaker than that in whole-animal assay, which was probably due to inadvertent loss of inflamed soft tissue when the joint was isolated. Fluorescence signals were hardly detected in lungs and hearts. Therefore although our PEI/siRNA complexes, like most siRNA nanoparticles, are subjected to clearance from the blood through RES and renal filtration, they also efficiently accumulate in the inflamed joints.

### *In vivo* cellular uptake of systemically administered PEI/siRNA complexes

**Figure 4:**
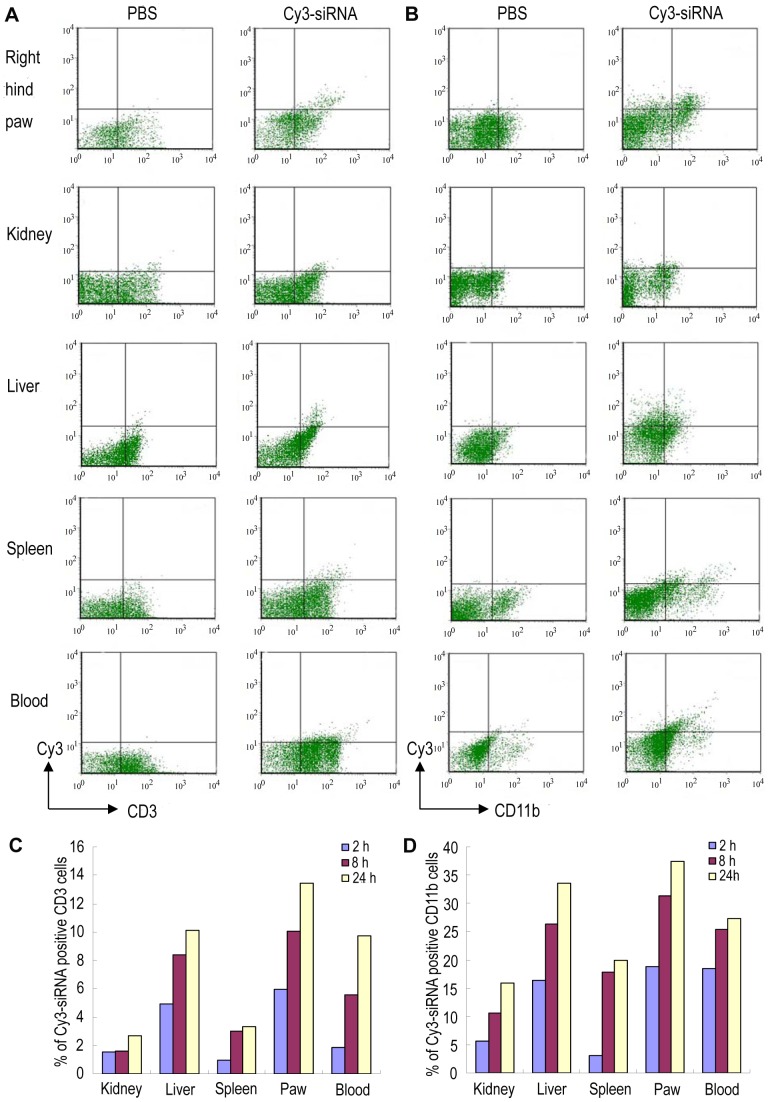
*In vivo* cellular uptake of siRNA polyplexes after single systemic administration. Arthritis rats were injected intravenously with 0.3/kg Cy3-siRNA formulated with PEI. The rat receiving phosphate buffered saline (PBS) was used as control. Cellular uptake of the Cy3-siRNA was evaluated by flow cytometry 2, 8, and 24 hours after injection using anti-CD3 and anti-CD11b mAbs. (A,B) Representative flow cytometry dot plots at 24 h. (C,D) Histograms showing percentages of Cy3-siRNA uptake within the gated CD3 or CD11b positive cells. Each value is an average of two determinations from two independent experiments. Data are representative of two independent experiments.

Two predominant cell types in the synovial infiltrate of RA, the phagocytic macrophages and the non-phagocytic T lymphocytes, were chosen to assess the preferential immune cell type targeted by PEI/siRNA particles. To this end, we collected blood, spleen, liver, kidney, and inflamed joints at three time points (2, 8, and 24 h post intravenous injection) from AA rats injected with PEI/Cy3-siRNA complexes and performed flow cytometry to examine the *in vivo* cellular uptake of the nanocomplexes. Representative flow cytometry staining for CD11b+ cells and CD3+ cells at 24 h post injection is presented in Figures 4A and B. Over the 24 h time course the proportion of CD11b- and CD3-positive cells positive for the fluorescence tended to gradually increase (Figures 4C, D). Although the PEI-encapsulated siRNA was detected in all of the tissues examined, it was preferentially taken up by macrophages and T cells in the paw. At 24 h, approximately 37% of CD11b+ cells and approximately 13% of CD3+ cells isolated from the paw had engulfed the Cy3-siRNA polyplexes. Note that at any time points in any tissues examined, CD11b+ cells took up siRNA polyplexes more efficiently than CD3+ cells. Lastly, it is worth mentioning that while kidney accumulates most PEI/siRNA particles as demonstrated by *in vivo* imaging, macrophages and T cells in this tissue seem less efficient in taking up the particles.

### Systemic delivery of PEI/IL-2/15Rβ siRNA nanoparticles inhibits inflammation in experimental arthritis

**Figure 5:**
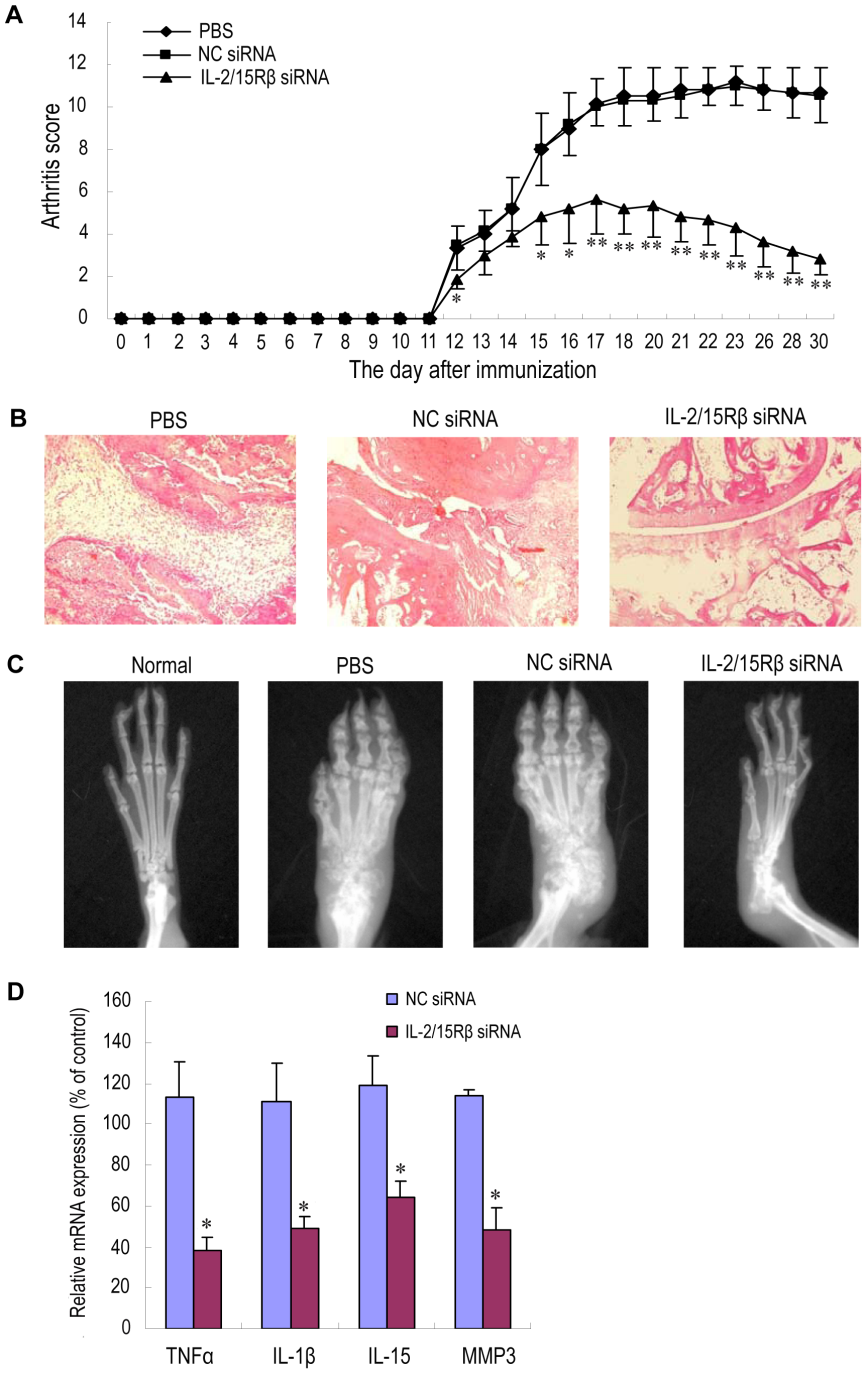
Therapeutic effects of PEI/IL-2/15Rβ siRNA complexes on AA rats. Male Wistar rats (n = 6 per group) were given Freund's complete adjuvant in the left hind footpad on day 0. IL-2/15Rβ siRNA-5 polyplexes were intravenously administered on days 10, 17, and 24 at 0.3 mg siRNA/kg. The rats receiving NC siRNA polyplexes or PBS were served as controls. (A) Arthritis score. Values are the mean and SD. *P<0.05; **P<0.01, versus the PBS group. (B) Representative histopathologies of the right hind ankle joints stained with hematoxylin and eosin (H&E). On day 31, rats were sacrificed and subjected to histopathological examination. Minimal articular inflammation and joint destruction were observed in the group treated with IL-2/15Rβ siRNA polyplexes, whereas PBS- and NC siRNA-treated rats exhibited pronounced synovial hyperplasia, bone damage and joint space narrowing. (C) Representative radiographs of right hind paws (n = 3 per group). On day 31, the rats were anaesthetized and subjected to radiography. Neither paw swelling nor joint damage was observed in normal rats. Severe bone erosion was seen in AA rats treated with either PBS or NC siRNA polyplexes, but the damage was much less in rats treated with IL-2/15Rβ siRNA polyplexes. (D) Effects of IL-2/15Rβ siRNA polyplexes on mRNA expression of inflammatory factors. At the end of the experiment (day 31), RNA was prepared from the right hind ankle joints of AA rats in each group. mRNA levels of each factor were determined by qPCR, normalized against GAPDH, and expressed as percentage of the PBS control. The result is presented as values (the mean and SD) obtained from three different samples randomly selected in each group. *P<0.05, versus PBS control.

To evaluate the *in vivo* efficacy of silencing IL-2/15Rβ for RA treatment, AA rats were intravenously injected with PEI/IL-2/15Rβ siRNA once per week for three weeks. Compared with phosphate-buffered saline (PBS) and NC siRNA control groups, administration of IL-2/15Rβ siRNA nanoparticles significantly reduced clinical signs of arthritis, as assessed by paw swelling and arthritis scores (Figure 5A). The maximum effect was observed at the end of the observation period. To objectively measure the extent of joint destruction, rats were subjected to radiography on day 31, then killed, and paws were isolated for histological evaluation of the ankle joints. Histological and radiographic examination confirmed these findings, showing that the reductions in synovial tissue inflammation and in cartilage/bone destruction were striking in the group treated with IL-2/15Rβ siRNA (Figures 5B, C).

To assess the effect of silencing IL-2/15Rβ on the expression of proinflammatory mediators in the arthritic joints, RNA was prepared from ankle joints of AA rats receiving different treatment, and cytokine expression was analyzed by qPCR. As shown in Figure 5D, expression of two well-known inflammatory cytokines TNFα and IL-1β in the group receiving IL-2/15Rβ siRNA decreased sharply as compared with NC siRNA-treated controls. Reduction in the transcript for MMP-3, which participates in cartilage/bone digestion, was also observed. Interestingly, reduction in IL-15 mRNA production upon Il-2/15Rβ knockdown was statistically significant but not so drastic compared with the reduced expression of TNFα, IL-1β, and MMP-3.

## Discussion

Previously we showed that immunotoxins targeting IL-15R-bearing cells (e.g. activated macrophages and T cells) can attenuate disease severity in rats with AA [15]. In view of the toxic moiety of immunotoxins and a wide distribution of IL-15R in multiple tissues, frequent administration of the immunotoxins may cause systemic side effects. It has been reported that siRNA can ensure a therapeutic effect for several weeks in non-dividing cells such as tissue macrophages [16], thus we believe inhibiting activation of IL-15R+ cells via siRNA-mediated gene silencing would be safer than eliminating IL-15R+ cells by cytotoxic protein agents. In this study we assessed the therapeutic efficiency of IL-2/15Rβ siRNA for systemic delivery in experimental arthritis. As expected, administration of IL-2/15Rβ siRNA decreased disease progression in AA rats, with striking inhibition of the clinical, radiologic, and histologic features of RA.

Because naked siRNA is subject to degradation by endogenous enzymes, and is too large and too negatively charged to freely cross cellular membranes, the issue of effective and non-toxic delivery is a key challenge and serves as the most significant barrier between siRNA technology and its therapeutic application [22]. It is widely recognized that viral vectors are highly efficient delivery systems for nucleic acids, but their clinical application is hindered by their induction of toxic immune responses and inadvertent gene expression changes following random integration into the host genome [23]. Cationic polymers, such as PEI, poly-L-lysine, and chitosan, can bind and condense large nucleic acids into stabilized nanoparticles and are used to be polymeric gene transfection materials as promising alternatives of viral vectors. Among them, PEI, which has strong escape capacity from the endosome due to the so-called “proton sponge” effect, is usually a gold standard of polymeric transfection agent [24]. We therefore chose to use *in vivo*-jetPEI, a linear PEI-based transfection reagent for *in vivo* experiments, as IL-2/15Rβ siRNA delivery vehicle. It has been reported that *in vivo*-jetPEI-formulated siRNA at N/P ratio of 8 does not induce inflammation and toxicity after systemic delivery [25]. In the future we plan to evaluate potential toxicity of the PEI/siRNA complexes at N/P ratio of 6 used in the present study.

There are few studies of systemic application of anticytokine siRNA by the intravenous route as a therapeutic tool in RA [26]–[28]. IL-15 contributes to the pathogenesis of arthritis by maintaining the activation of synovial macrophages which in turn are responsible for the production and release of most proinflammatory factors, including IL-15 itself. The observed therapeutic effect of IL-2/15Rβ siRNA on AA rats was associated with decreased expression of proinflammatory mediators in the ankle joints, including TNFα, IL-1β, MMP-3, and IL-15. Compared with marked influence on the expression of TNFα, IL-1β, and MMP-3, reduction in IL-15 mRNA production upon IL-2/15Rβ knockdown was not so drastic, consistent with the role of IL-15 signaling in the amplification of the inflammatory network in RA. Because TNFα is a major proflammatory cytokine in RA, and it is IL-15, not IL-2, that induces the production of abundant TNFα by synovial macrophages and T cells [4], we speculate while IL-2/15Rβ is a common signaling subunit shared by IL-2 and IL-15, the antiarthritic effect of IL-2/15Rβ siRNA may be primarily ascribed to the inhibition of IL-15 signaling. This view is strengthened by the fact that IL-15, not IL-2, plays a role in the development of osteoclasts which were reported to be involved in the pathological destruction of bone [29]. On the other hand however, IL-2, like IL-15, may also stimulate the proliferation of T cells, B cells, and NK cells [30] that all participate in RA pathogenesis, the antiarthritic effect of IL-2/15Rβ siRNA can thus be secondarily attributed to the inhibition of IL-2 signaling.

In tissue biodistribution experiments, PEI/siRNA complexes showed greater selective accumulation in kidney and mild or intermediate incorporation by liver and spleen. This is consistent with classic clearance of a majority of intravenously administered foreign particles by renal filtration and RES uptake. It has been reported that siRNA is barely detectable in normal joints and is increased in the inflamed joints of arthritic mice [26]. Similarly, in this study significant amounts of PEI/siRNA particles were found to accumulate in the inflamed joints, which was most likely resulted from the enhanced permeability and retention (EPR) effect due to the abnormal and leaky vasculature in the joints of RA. Thus, although further studies will be necessary, based on imaging data, the PEI/siRNA complexes are preferentially retained in kidney 12 h after intravenous injection, followed by liver, joints, and spleen, whereas accumulation is barely detectable in heart and lung. Carriers such as liposomes, drug-polymer conjugates, and PLGA nanoparticles have been utilized for EPR effect-based passive targeting to the inflamed synovium [26], [27], [31]. High uptake of the PEI/siRNA complexes by arthritic paws confirms that PEI-mediated siRNA delivery has the potential for the treatment of RA.

Nanoparticles experience rapid clearance by the kidney if they are smaller than 10 nm in diameter [32], [33]. In addition, the physicochemical characteristics of nanoparticles such as particle size and surface charge can dramatically affect clearance mechanism via glomerular basement membrane (GBM)-mediated disruption of siRNA nanoparticles. It has been recently suggested that cationic polymer-based siRNA nanoparticles being positive in zeta potential and being ≈100 nm or smaller in hydrodynamic diameters can deposit and disassemble at the kidney GBM [34], [35]. Considering the size of our PEI/siRNA particles (average diameter 246 nm), it is unlikely that they could access the GBM and disassemble there to generate components that are small enough to cross into the urinary space. Therefore, although PEI/siRNA nanoparticles are primarily accumulated in kidney over a period of 12 h after administration, renal filtration does not seem to be the main elimination pathway for the clearance of these particles from circulation. To gain a more complete picture of tissue uptake and elimination pathway of jetPEI/siRNA particles, additional studies over a longer time course as well as assessment of tissues (including urine) no studied here are needed.

A critical requirement for achieving *in vivo* RNA interference using a systemic approach is to ensure the delivery of siRNA to the cytoplasm of the cell. In the present study, a significant proportion of macrophages in the inflamed paws ingested Cy3-siRNA polyplexes following intravenous injection, consistent with marked alleviation of disease severity in AA rats treated with IL2/15Rβ siRNA polyplexes. In addition, considering that RA is a systemic inflammatory disease, manifested locally as erosion of the joints, and that the pathogenesis of RA involves systemic-derived cellular infiltration and cytokine production [36], [37], it is possible that CD11b+ cells transfected by PEI/IL2/15Rβ siRNA particles in other places might migrate to inflamed joints to counterbalance local inflammation. Besides avid uptake of the particles by macrophages, a notable proportion of T cells isolated from the paws also entrapped the siRNA, which could be attributed to the surface charge of the PEI/ siRNA complexes. Perhaps T cells, being nonphagocytic cells, prefer ingesting cationic particles [38]. Since both macrophages and T cells are involved in the pathogenesis of RA, systemic inhibition of macrophage/T cell activation via downregulating IL-15 and IL-2 signaling may result in an enhanced anti-arthritic effect. It has been recently reported that a combined therapy with anti-CD3 and anti-TNF leads to a long-term amelioration of established arthritis in an animal model [39].

In conclusion, PEI-complexed IL-2/15Rβ siRNA particles are capable of downregulating IL-2/15Rβ *in vitro* in difficult-to-silence peritoneal macrophages from rats. *In vivo* experiments demonstrate that the siRNA polyplexes can easily accumulate in arthritic paws and a significant proportion of macrophages and T cells in the target tissue can take up the particles. As expected, weekly administered PEI-complexed IL-2/15Rβ siRNA is effective in reducing disease severity in AA rats, confirming that IL-2/15Rβ could be therapeutically targeted for the treatment of RA.

## Materials and Methods

### Ethics statement

All animal experiments were performed in accordance with the guideline of and approved by the Committee on Laboratory Animals of Southeast University, China. All surgery and in vivo imaging were performed under sodium pentobarbital anesthesia, and all efforts were made to minimize suffering.

### siRNA duplexes

Six siRNA duplexes directed against rat IL-2/15Rβ and a negative control (NC) siRNA were synthesized by GenePharma (Shanghai, China). The sense sequences for each of the IL-2/15Rβ siRNA were as follows: siRNA-1, 5′CGGAGAUGUAACAUAAGCUTT; siRNA-2, 5′GAGGAUGCAUCCGUAUUCATT; siRNA-3, 5′GGAAGUGCUUGACAGAGAUTT; siRNA-4, 5′GCCUAUGGGAACAGCAUAATT; siRNA-5, 5′GAAGGGAUGUCUACCAAUATT; siRNA-6, 5′GGGAUGGGAAGGAUCAUAATT. To assess silencing efficiency, siRNA duplexes (40 nM) were separately transfected into rat peritoneal macrophages using X-tremeGENE siRNA transfection reagent (Roche). After 24 h, total RNA was extracted using TRIzol extraction (Takara, Japan) according to the manufacturer's instructions. Then RNA was reverse-transcribed into cDNA with a mix of oligo dT and random primers. SYBR green (Takara, Japan) based quantitative real-time PCR (qPCR) was performed to measure the silencing of IL-2/15Rβ mRNA using GAPDH as an endogenous control. The primer pairs for IL-2/15Rβ and GAPDH were: IL-2/15Rβ forward, 5′CTTCTTGTCCTGCGTCTG; reverse, 5′GGATGTGGCACTTGAGAA; GAPDH forward, 5′GCAAGAGAGAGGCCCTCAG; reverse 5′TGTGAGGGAGATGCTCAGTG. The PCR reaction was performed at 95°C for 30 s, followed by 40 cycles of 95°C for 5 s and 60°C for 34 s. IL-2/15Rβ expression was normalized to GAPDH and calculated as the percentage of NC siRNA. The most potent siRNA was further confirmed by Western blotting.

NC siRNA labeled with Cy3 or Cy5 (Cy3-siRNA, Cy5-siRNA) was purchased from RiboBio (Guangzhou, China).

### Preparation of PEI/siRNA complexes

*In vivo*-jetPEI was ordered from PolyPlus Transfection. PEI/siRNA complexes were formed at an N/P ratio of 6 following the recommendations of the manufacturer.

### Properties of PEI/siRNA complexes

The particle size and surface charge of PEI/siRNA complexes (N/P ratio of 6) were measured by dynamic light scattering (DLS).

### Peritoneal macrophage isolation and cell culture

Rat peritoneal macrophages were isolated by adhesion to plastic culture dishes 3 days after intraperitoneal injection of 4% thioglycollate media (Sigma). They were cultured at 37°C in high glucose DMEM medium (Gibco) containing 10% fetal bovine serum (FBS), 100 U/mL penicillin, and 100 µg/mL streptomycin.

### Western blot analysis

Rat peritoneal macrophages were incubated with 40 nM siRNA within PEI/siRNA nanoparticles or transfected with siRNA using X-tremeGENE siRNA transfection reagent. 48 h after transfection, cells were harvested and lysed on ice in lysis buffer containing 50 mM Hepes (pH 7.5), 100 mM NaCl, 1 mM EDTA, 10% glycerol, 1% NP-40, and 0.5 mM PMSF. Cellular debris was removed and protein was collected by centrifugation. Protein concentrations in the extracts were measured with Pierce BCA protein assay kit. Equal amounts (30 µg) of protein were resolved by 10% SDS-PAGE and electrotransferred to PVDF membranes. Then the membranes were blocked in 2.5% bovine serum albumin (BSA)-TBST (1.0 M Tris-Cl, pH 8.0, 150 mM NaCl, 0.05% Tween-20), incubated with rabbit anti-IL-2Rβ (M-20) or anti-β actin (R-22) antibodies (Santa Cruz Biotechnology, USA) at a dilution of 1∶2000, and followed by probing with horseradish peroxidase-labeled goat anti-rabbit antibody (Wuhan Boster Biological Technology, China).

### Arthritis induction and treatment

All animal experiments were done following the guidelines approved by the Committee on Animals of Southeast University, China. Wistar male rats (specific pathogen-free) were purchased from Shanghai Laboratory Animal Center of Chinese Academy of Sciences. Rats were housed in a clean pathogen-free environment. To produce adjuvant-induced arthritis (AA), the footpad of the left hind paw was injected intradermally with 100 µL Freund's complete adjuvant (CFA) containing 10 mg/mL heat-killed Bacille Calmette-Guerin (BCG) freeze-dried powder (National Institutes for Food and Drug Control, China) in sterile liquid paraffin on day 0. About 11 days after immunization arthritis was developed in CFA-uninjected paws of some rats, and by day 13 AA was induced in all immunized rats.

For therapeutic treatment studies, from day 10 on, rats with ongoing arthritis in the treatment group (n = 6) were injected intravenously with PEI/IL-2/15Rβ siRNA complexes (0.3 mg/kg siRNA) once a week for three weeks (on days 10, 17, and 24). The other 2 groups (n = 6) were injected with phosphate buffered saline (PBS) or PEI/NC siRNA, respectively.

Arthritis in each paw was scored as previously described [15]. Three paws except the CFA-injected left hind paw were scored, so the highest possible score per rat was 12.

### *In vitro* cellular uptake of PEI/siRNA complexes

For *in vitro* cellular uptake studies, rat peritoneal macrophages were seeded in 24-well plates at 2×10^5^ cells/1.88 cm^2^. Cy3-siRNA-loaded PEI with an N∶P ratio of 6 were incubated with macrophages for 12 h. Then the cells were trypsinized into single cell suspension with 0.25% trypsin-EDTA, collected by centrifugation at 1000 rpm for 10 min, washed three times with PBS, and resuspended in 500 µL of PBS containing 1% FBS. Intracellular uptake was analyzed by flow cytometry. The transfection efficiency was calculated as the percentage of the fluorescence- emitting cells in the total number of cells.

### *In vivo* imaging

AA rats that had developed overt arthritis with a mean clinical score of at least 9 (three paws except the CFA-injected paw) were used for *in vivo* imaging. Rats were randomly assigned to three groups (n = 3 per group). PEI/Cy5-siRNA nanoparticles were injected as a single dose (0.6 mg siRNA/kg) via tail vein. AA rats that did not receive PEI/Cy5-siRNA served as control. The rats were anaesthetized by isoflurane inhalation. At different time points after injection, the *in vivo* images were observed with Caliper IVIS Spectrum imaging system (excitation 640 nm, emission 680 nm) and recorded by a built-in CCD camera. After 12 h, the rats were killed, and the excised paws and major organs were also imaged.

### *In vivo* cellular uptake of PEI/siRNA complexes

Rats that had developed overt arthritis with a mean clinical score of at least 9 were used. The rats were injected intravenously with a single dose of Cy3-siRNA (0.3 mg/kg) formulated with PEI. As controls, other rats were injected with PBS. Blood, liver, spleen, kidney, and paws were harvested at 3 time points (2, 8, and 24 h post injection). Mononuclear cells from blood and single cell suspensions from liver, spleen, and kidney were prepared using standard procedures. For cell isolation from arthritic paws, right hind ankle joints were isolated and digested with 1 mg/mL of collagenase D (Roche) for 60 min at 37°C. Isolated cells were stained with anti-rat CD11b PerCP-eFluor® 710 or anti-rat CD3 FITC (eBioscience, San Diego, CA) for 20 min at 4°C and analyzed by flow cytometry. The percentage of CD11b+ or CD3+ cells positive for Cy3 fluorescence was given as the percentage of cells in the upper right and lower right quadrants.

### Histologic analysis

Right hind paws were resected above the ankle joint, fixed in 10% phosphate buffered formaldehyde solution, and decalcified in 10% EDTA. The joints were then dehydrated, embedded in paraffin and sectioned longitudinally. Serial 4-µm sections of ankle joints were stained with hematoxylin and eosin (H&E).

### Radiographic assessment

On day 31, three rats from each group were selected at random and anesthetized by intraperitoneal injection of sodium pentobarbital. Normal rats were used as control. Radiographs of the right hind paws were obtained with a lumina XR system (Caliper IVIS Spectrum).

### Quantitation of mRNA for proinflammatory cytokines

Right hind ankle joint were isolated by removing the skin and then stored at −80°C until use. Total RNA was obtained using TRIzol extraction (Takara, Japan) according to the manufacturer's instructions (n = 3 in each group). The RNA was reverse transcribed into complementary DNA (cDNA) with a mix of oligo dT and random primers. The mRNA amounts for TNFα, IL-1β, IL-15, and MMP3 were quantified by qPCR as described previously [15]. The PCR reaction was performed at 95°C for 30 s, followed by 40 cycles of 95°C for 5 s and 60°C for 34 s. Cytokine expression was normalized to GAPDH and presented as percentage of the PBS controls.

### Statistics

Statistical analyses were performed using the non-parametric one-way ANOVA. The results were shown as mean ± SD.

## References

[1] Brennan FM, McInnes IB (2008) Evidence that cytokines play a role in rheumatoid arthritis. J? Clin Invest 118: 3537–3545. 18982160 10.1172/JCI36389 PMC2575731

[2] McInnes IB, O'Dell JR (2010) State-of-the-art: rheumatoid arthritis. Ann Rheum Dis 69: 1898–1906. 20959326 10.1136/ard.2010.134684

[3] McInnes IB, al-Mughales J, Field M, Leung BP, Huang FP, et al (1996) The role of interleukin-15 in T-cell migration and activation in rheumatoid arthritis. Nat Med 2: 175–182. 8574962 10.1038/nm0296-175

[4] McInnes IB, Leung BP, Sturrock RD, Field M, Liew FY (1997) Interleukin-15 mediates T cell-dependent regulation of tumor necrosis factor-alpha production in rheumatoid arthritis. Nat Med 3: 189–195. 9018238 10.1038/nm0297-189

[5] Asquith DL, McInnes IB (2007) Emerging cytokine targets in rheumatoid arthritis. Curr Opin Rheumatol 19: 246–251. 17414950 10.1097/BOR.0b013e3280eec78c

[6] McInnes IB, Gracie JA (2004) Interleukin-15: a new cytokine target for the treatment of inflammatory diseases. Curr Opin Pharmacol 4: 392–397. 15251134 10.1016/j.coph.2004.04.003

[7] Waldmann TA, Tagaya Y (1999) The multifaceted regulation of interleukin-15 expression and the role of this cytokine in NK cell differentiation and host response to intracellular pathogens. Annu Rev Immunol 17: 19–49. 10358752 10.1146/annurev.immunol.17.1.19

[8] Ernestam S, af Klint E, Catrina AI, Sundberq E, Enqstrom M, et al (2006) Synovial expression of IL-15 in rheumatoid arthritis is not influenced by blockade of tumour necrosis factor. Arthritis Res Ther 8: R18. 16507118 10.1186/ar1871 PMC1526582

[9] Andersson AK, Feldmann M, Brennan FM (2008) Neutralizing IL-21 and IL-15 inhibits pro-inflammatory cytokine production in rheumatoid arthritis. Scand? J? Immunol 68: 103–111. 18482208 10.1111/j.1365-3083.2008.02118.x

[10] González-Álvaro I, Ortiz AM, Alvaro-Gracia JM, Castañeda S, Díaz-Sánchez B, et al (2011) Interleukin 15 levels in serum may predict a severe disease course in patients with early arthritis. PLoS One 6(12): e29492. 22242124 10.1371/journal.pone.0029492 PMC3248461

[11] Knevel R, Krabben A, Brouwer E, Posthumus MD, Wilson AG, et al (2012) Genetic variants in IL15 associate with progression of joint destruction in rheumatoid arthritis: a multicohort study. Ann Rheum Dis 71: 1651–1657. 22440823 10.1136/annrheumdis-2011-200724

[12] Schett G, Gravallese E (2012) Bone erosion in rheumatoid arthritis: mechanisms, diagnosis and treatment. Nat Rev Rheumatol 8: 656–664. 23007741 10.1038/nrrheum.2012.153 PMC4096779

[13] Ruchatz H, Leung BP, Wei XQ, McInnes IB, Liew FY (1998) Soluble IL-15 receptor alpha-chain administration prevents murine collagen-induced arthritis: a role for IL-15 in development of antigen-induced immunopathology. J? Immunol 160: 5654–5660. 9605172

[14] Ferrari-Lacraz S, Zanelli E, Neuberg M, Donskoy E, Kim YS, et al (2004) Targeting IL-15 receptor-bearing cells with an antagonist mutant IL-15/Fc protein prevents disease development and progression in murine collagen-induced arthritis. J? Immunol 173: 5818–5826. 15494535 10.4049/jimmunol.173.9.5818

[15] Wang D, Deng X, Leng X, Mao X (2010) Interleukin-15 receptor-directed immunotoxins atteunuate disease severity in rat adjuvant arthritis. Mol Immunol 47: 1535–1543. 20188418 10.1016/j.molimm.2010.01.023

[16] Bartlett DW, Davis ME (2006) Insights into the kinetics of siRNA-mediated gene silencing from live-cell and live-animal bioluminescent imaging. Nucleic Acids Res 34: 322–333. 16410612 10.1093/nar/gkj439 PMC1331994

[17] Fehniger TA, Caligiuri MA (2001) Interleukin 15: biology and relevance to human disease. Blood 97: 14–32. 11133738 10.1182/blood.v97.1.14

[18] Schindler CW (2002) Series introduction. JAK-STAT signaling in human disease. J? Clin Invest 109: 1133–1137. 11994400 10.1172/JCI15644 PMC150971

[19] Kurreeman FA, Daha NA, Chang M, Catanese JJ, Begovich AB, et al (2009) Association of IL2RA and IL2RB with rheumatoid arthritis: a replication study in a Dutch population. Ann Rheum Dis 68: 1789–1790. 19822714 10.1136/ard.2008.106393

[20] Kuuliala A, Nissinen R, Kautiainen H, Repo H, Leirisalo-Repo M (2006) Low circulating soluble interleukin 2 receptor level predicts rapid response in patients with refractory rheumatoid arthritis treated with infliximab. Ann Rheum Dis 65: 26–29. 15941839 10.1136/ard.2004.034728 PMC1797973

[21] Gao J, Liu W, Xia Y, Li W, Sun J, et al (2011) The promotion of siRNA delivery to breast cancer overexpressing epidermal growth factor receptor through anti-EGFR antibody conjugation by immunoliposomes. Biomaterials 32: 3459–3470. 21296406 10.1016/j.biomaterials.2011.01.034

[22] Whitehead KA, Langer R, Anderson DG (2009) Knocking down barriers: advances in siRNA delivery. Nat Rev Drug Discov 8: 129–138. 19180106 10.1038/nrd2742 PMC7097568

[23] Akhtar S, Benter IF (2007) Nonviral delivery of synthetic siRNAs in vivo. J? Clin Invest 117: 3623–3632. 18060020 10.1172/JCI33494 PMC2096447

[24] Guo S, Huang Y, Jiang Q, Sun Y, Deng L, et al (2010) Enhanced gene delivery and siRNA silencing by gold nanoparticles coated with charge-reversal polyelectrolyte. ACS Nano 4: 5505–5511. 20707386 10.1021/nn101638u PMC3044603

[25] Bonnet ME, Erbacher P, Bolcato-Bellemin AL (2008) Systemic delivery of DNA or siRNA mediated by linear polyethylenimine (L-PEI) does not induce an inflammatory response. Pharm Res 25: 2972–2982. 18709489 10.1007/s11095-008-9693-1

[26] Khoury M, Louis-Plence P, Escriou V, Noel D, Largeau C, et al (2006) Efficient new cationic liposome formulation for systemic delivery of small interfering RNA silencing tumor necrosis factor alpha in experimental arthritis. Arthritis Rheum 54: 1867–1877. 16729293 10.1002/art.21876

[27] Khoury M, Escriou V, Courties G, Galy A, Yao R, et al (2008) Efficient suppression of murine arthritis by combined anticytokine small interfering RNA lipoplexes. Arthritis Rheum 58: 2356–2367. 18668557 10.1002/art.23660

[28] Ye C, Bhan A, Deshpande V, Shankar P, Manjunath N (2013) Silencing TNF-α in macrophages and dendritic cells for arthritis treatment. Scand? J? Rheumatol 42: 266–269. 23582054 10.3109/03009742.2013.777779

[29] Ogata Y, Kukita A, Kukita T, Komine M, Miyahara A, et al (1999) A novel role of IL-15 in the development of osteoclasts: inability to replace its activity with IL-2. J? Immunol 162: 2754–2760. 10072521

[30] Bachmann MF, Oxenius A (2007) Interleukin 2: from immunostimulation to immunoregulation and back again. EMBO Rep 8: 1142–1148. 18059313 10.1038/sj.embor.7401099 PMC2267244

[31] Mitragotri S, Yoo JW (2011) Designing micro- and nano-particles for treating rheumatoid arthritis. Arch Pharm Res 34: 1887–1897. 22139688 10.1007/s12272-011-1109-9

[32] Choi HS, Liu W, Misra P, Tanaka E, Zimmer JP, et al (2007) Renal clearance of quantum dots. Nat Biotechnol 25: 1165–1170. 17891134 10.1038/nbt1340 PMC2702539

[33] Choi HS, Ipe BI, Misra P, Lee JH, Bawendi MG, et al (2009) Tissue- and organ-selective biodistribution of NIR fluorescent quantum dots. Nano Lett 9: 2354–2359. 19422261 10.1021/nl900872r PMC2782558

[34] Zuckerman JE, Choi CH, Han H, Davis ME (2012) Polycation-siRNA nanoparticles can disassemble at the kidney glomerular basement membrane. Proc Natl Acad Sci? U? S? A 109: 3137–3142. 22315430 10.1073/pnas.1200718109 PMC3286910

[35] Naeye B, Deschout H, Caveliers V, Descamps B, Braeckmans K, et al (2013) In vivo disassembly of IV administered siRNA matrix nanoparticles at the renal filtration barrier. Biomaterials 34: 2350–2358. 23261216 10.1016/j.biomaterials.2012.11.058

[36] Howard KA, Paludan SR, Behlke MA, Besenbacher F, Deleuran B, et al (2009) Chitosan/siRNA nanoparticle-mediated TNF-alpha knockdown in peritoneal macrophages for anti-inflammatory treatment in a murine arthritis model. Mol Ther 17: 162–168. 18827803 10.1038/mt.2008.220 PMC2834976

[37] Arend WP (2001) Physiology of cytokine pathways in rheumatoid arthritis. Arthritis Rheum 45: 101–106. 11308054 10.1002/1529-0131(200102)45:1<101::AID-ANR90>3.0.CO;2-7

[38] Fröhlich E (2012) The role of surface charge in cellular uptake and cytotoxicity of medical nanoparticles. Int? J? Nanomedicine 7: 5577–5591. 23144561 10.2147/IJN.S36111 PMC3493258

[39] Dépis F, Hatterer E, Lamacchia C, Waldburger JM, Gabay C, et al (2012) Long-term amelioration of established collagen-induced arthritis achieved with short-term therapy combining anti-CD3 and anti-tumor necrosis factor treatments. Arthritis Rheum 64: 3189–3198. 22508436 10.1002/art.34497

